# Dendritic Cells Are Associated with Prognosis and Survival in Breast Cancer

**DOI:** 10.3390/diagnostics11040702

**Published:** 2021-04-14

**Authors:** Joanna Szpor, Joanna Streb, Anna Glajcar, Paulina Frączek, Aleksandra Winiarska, Katarzyna E. Tyrak, Paweł Basta, Krzysztof Okoń, Robert Jach, Diana Hodorowicz-Zaniewska

**Affiliations:** 1 Department of Pathomorphology, Jagiellonian University Medical College, 31-531 Kraków, Poland; joanna.szpor@uj.edu.pl (J.S.); aglajcar@su.krakow.pl (A.G.); winiarskaaleksandra0706@gmail.com (A.W.); k.okon@uj.edu.pl (K.O.); 2 Department of Oncology, Jagiellonian University Medical College, 31-531 Kraków, Poland; paulinafraczek2@gmail.com; 3 Internal Medicine, Faculty of Medicine, Jagiellonian University Medical College, 31-066 Kraków, Poland; katarzyna.tyrak@uj.edu.pl; 4 Department of Gynecology and Oncology, Jagiellonian University Medical College, 31-501 Kraków, Poland; pawel.basta@uj.edu.pl (P.B.); jach@cm-uj.krakow.pl (R.J.); 5 General, Oncological, and Gastrointestinal Surgery, Jagiellonian University Medical College, 30-688 Kraków, Poland; diana.hodorowicz-zaniewska@uj.edu.pl

**Keywords:** dendritic cells, breast cancer, molecular subtype

## Abstract

Dendritic cells (DCs) constitute a part of the tumour microenvironment, but we are still far from understanding their complex role in immune response to the tumour. This study aimed to investigate the density of DCs expressing CD1a, CD83, CD123, DC-LAMP3 (CD208) and DC-SIGN (CD209) in breast cancer. The correlations between DC density and molecular subtype of breast cancer, its hormone receptor status, spatial location and their associations with clinical and pathological prognostic factors were evaluated. We have shown that intratumoural CD1a+ cells were significantly associated with progression-free survival. For LAMP3+ and CD123+ DCs, higher cell densities were associated with non-luminal as compared to luminal cancer phenotype. In contrast, dense CD83+ DC infiltrate was observed in luminal tumours. The number of CD1a+ DCs in both locations was the highest in luminal B/HER2+ cancers. The highest positive cell count of LAMP3+ cells was observed in the triple-negative subtype in both locations. We found higher numbers of LAMP3+ DCs both intratumourally and at the invasive margin, as well as CD123+ DCs intratumourally in tumours with negative expression of oestrogen or progesterone receptors. Our study demonstrates associations between DC subpopulations and histological and clinical characteristics, as well as molecular subtypes in breast carcinoma.

## 1. Introduction

Breast cancer attracts widespread interest as it is the leading malignant neoplasm in women and one of the most common causes of cancer death worldwide. It is a very heterogeneous disease, both clinically and molecularly, comprising various subtypes with distinct biological characteristics and response to therapy. Although it is widely considered to be a poorly immunogenic tumour [1,2], like every human malignant neoplasm, breast cancer induces an immune response in its microenvironment. Recent studies have shed new light on immune cells as important prognostic and predictive biomarkers in this malignancy [3]. Among them, dendritic cells (DCs) seem to play a crucial role in tumour-associated immunological reactions, acting as the most potent antigen-presenting cells. DCs recognise, process and present tumour-derived antigens in the context of major histocompatibility complexes (MHCs), and subsequently trigger a naïve T cell response, linking innate and adaptive immunity [1,4]. Immature DCs, stemming from bone marrow precursors, show high endocytic activity and a low level of T lymphocytes activation, so they probably encourage antigen-specific tolerance rather than immunity. Upon contact with the antigen, these immature DCs are activated into mature DCs, which migrate to the lymphoid organs where they activate T lymphocytes, thus generating an antigen-specific response [1]. Since the population of DCs is heterogeneous and remarkably plastic in its immunoregulatory potential, DCs can have either a positive or negative effect on host immunity [5]. Depending on the type, level of maturation as well as functional state of the DCs, they can increase effector T cell responses (favouring better outcomes) or mediate T cell tolerance (ensuing tumour progression) [4,5].

Researchers who studied the population of dendritic cells in various human malignancies have shown that the increased number of tumour-infiltrating DCs is in general associated with improved survival and decreased recurrence rates in cancer patients [6,7,8,9]. However, high infiltration of plasmacytoid DCs (pDCs) in breast cancer seems to correlate with an adverse clinical outcome [10,11]. Additionally, the importance of DC maturation state in malignant lesions was confirmed by studies showing a positive association between the number of mature DCs in the tumour area and a favourable prognosis [12,13]. Nonetheless, despite the pivotal role attributed to DCs in the development and propagation of various cancers, we are still far from understanding the complete nature and role of these cells as prognostic and predictive biomarkers in breast carcinoma.

The aim of the present study was to investigate the density of DCs expressing CD1a, CD83, CD123, DC-LAMP3 (CD208) and DC-SIGN (CD209) in breast cancers. CD1a is a non-classical MHC class I antigen [14]. CD1a is often used as a marker of immature dendritic cells, but it is expressed on both immature as well as mature DCs [15]. The activation status of DCs is based on the expression of CD83, DC-SIGN and DC-LAMP3, which are nonspecific indicators of mature DCs [2,4,5]. CD123 is the marker of immature plasmacytoid dendritic cells [4,16,17]. In our study, the correlations between DC density and molecular subtype of breast cancer, its hormone receptor status, spatial location as well as their associations with clinical and pathological prognostic factors were evaluated. The obtained results might provide clues as to the prognostic and predictive significance of tumour-infiltrating dendritic cells, as well as prove useful for the design of potential therapeutic strategies in breast cancer patients.

## 2. Materials and Methods

The material consisted of routinely processed, formalin-fixed paraffin-embedded primary invasive breast carcinomas diagnosed between 2002 and 2014. The archival haematoxylin–eosin-stained slides were re-evaluated and representative, well-preserved specimens were chosen for immunohistochemistry. For nuclear grading, the Nottingham Histologic Grade system was used, while staging was performed according to the 2017 American Joint Committee on Cancer system [18].

Immunohistochemistry (IHC) for: CD1a, CD123, CD83, DC-LAMP and DC-SIGN, oestrogen receptor (ER), progesterone receptor (PR), Ki67 and HER2 was performed according to the protocol routinely used in our laboratory as previously described [19]. Primary antibodies, dilution as well as the retrieval procedure used in our study are summarized in Table 1.

The slides stained for DC markers were scanned on a Nikon Labophot-2 optical microscope (Tokyo, Japan) at low magnification (100×), and the areas with the highest number of positive cells were chosen. Then, positively stained cells were counted in 5 high power fields (HPF) (400×, 0.2 mm^2^ field area), which represented 1 mm^2^ of the examined tissue. The positive cells located no further than 1 HPF from the tumour edge were regarded as invasive margin, while positive cells located within neoplastic tissue, further than 1 HPF from the tumour edge inwards, were considered as intratumoural population.

Positive ER and PR expression were set when ≥1% of neoplastic cells showed positive immunostaining. The threshold for discriminating between low and high Ki67 expression was set at ≥20% of positive cells. Scoring of the HER2 stain was performed by standard method [20]. The cases were classified into molecular subtypes according to the 2015 St. Gallen International Expert Consensus [21,22].

To assess differences in positive cell infiltrate between groups, ANOVA Kruskal–Wallis, U Mann–Whitney and Wilcoxon tests were performed. The correlations between groups were evaluated by using the Spearman rank test. Survival analyses for progression-free survival (PFS) were estimated using the Kaplan–Meier method. The comparisons according to different variables were performed with a log-rank test. A multivariate Cox proportional hazard regression model was performed to examine the effect of independent factors on PFS. All of the ranges were described with 95% confidence interval (CI). All analyses were performed using Statistica 10 (StatSoft Inc., Tulsa, OK, USA). *p* values ≤ 0.05 were considered significant.

The study was approved by the Jagiellonian University Committee of Bioethics (consent number KBET/72/B/2014).

## 3. Results

### 3.1. Description of Study Group

The analysed group consisted of 152 cases diagnosed as primary invasive breast carcinoma. The patients and tumour characteristics are summarized in Table 2.

### 3.2. DC Subpopulations in Molecular Cancer Subtypes

First, we investigated whether the DC counts differed between cancers of luminal and non-luminal subtypes. Statistically significant differences were observed for LAMP3+ and CD123+ DCs, in either intratumoural location or at the invasive margin (*p* < 0.015 for each DC subpopulation), as well as for CD83+ cells of intratumoural area (*p* < 0.03). For LAMP3+ and CD123+ populations, higher cell densities were associated with non-luminal as compared to luminal cancer phenotype. In contrast, dense CD83+ DC infiltrate was observed in luminal tumours. Thorough analysis of each of these DC counts showed significant differences in the density of infiltration between molecular subtypes of breast cancer. The number of CD1+ DCs in both locations was the highest in luminal B/HER2+ cancers. DC count was significantly lower in luminal/HER2− than in luminal B/HER2+ subtype (*p* < 0.001), and higher in luminal B/HER2+ as compared to HER2+ non-luminal subtype (*p* < 0.03). Similar results were obtained for invasive margin CD1a+ cells, with *p* < 0.001 in the former and *p* < 0.01 in the latter pair of groups.

Regarding intratumoural LAMP3+ cells, the highest positive cell count was observed in the triple-negative subtype, which was significantly higher than in the luminal/HER2+ (*p* < 0.001) and luminal/HER2− (*p* < 0.001) subtype, followed by HER2+ non-luminal cancers, which contained significantly more LAMP3+ cells within the tumour than luminal/HER2− (*p* = 0.04) and luminal B/HER2+ (*p* < 0.015) subtypes. LAMP3+ DCs at the invasive margin were also the most abundant in triple-negative tumours; the difference was statistically significant in comparison to luminal/HE2R− (*p* = 0.02) and luminal/HER2+ (*p* < 0.001) cancers.

The intratumoural CD123+ cell number was the lowest in luminal/HER2− cancers; the difference was statistically significant in comparison to luminal/HER2+ (*p* < 0.006), HER2+ non-luminal (*p* < 0.001) and triple negative (*p* < 0.02) cancers. With regard to invasive margin CD123+ cells, the only significant association was observed between luminal/HER2− and triple-negative subtypes (*p* < 0.03), with a higher positive cell content in the latter group.

**Figure 1:**
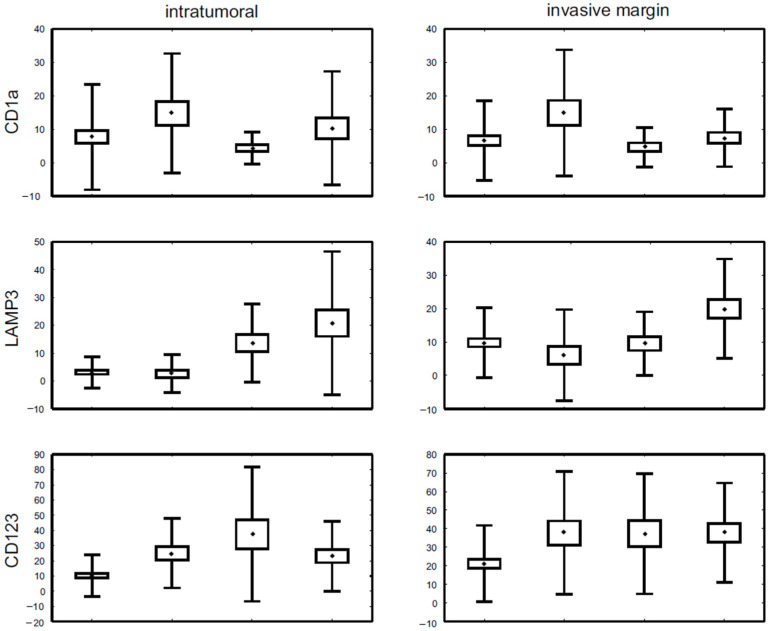

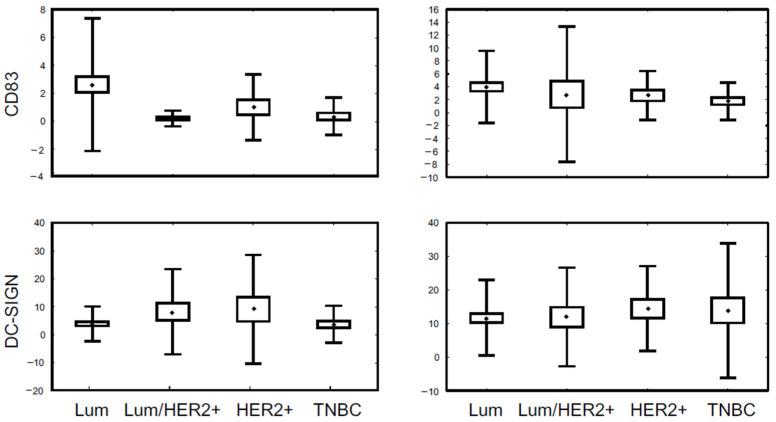
The density of investigated dendritic cell (DC) subpopulations in breast cancer specimens representing different molecular subtypes. Abbreviations used: Lum/HER2+—luminal/HER2+, HER2+—HER2+ non-luminal, TNBC—triple negative subtype. The central point is the arithmetic mean, the box is the arithmetic mean ± standard error and the whisker is the arithmetic mean ± standard deviation.

For intratumoural and invasive margin CD83+ DCs, the differences were statistically significant in the Kruskal–Wallis ANOVA test exclusively. In a pairwise comparisons test, no statistical significance concerning cell count was noted between any of the investigated molecular subtypes. The most pronounced differences in intratumoural CD83+ cell number were observed between luminal/HER2− and triple-negative (*p* < 0.15), while at the invasive margin CD83+ cells between luminal/HER2− and luminal/HER2+ (*p* < 0.15). Results are shown in Figure 1 and Table 3. As we were not able to show significant differences in particular DC populations between luminal A and luminal B/HER2− subtypes (data not shown), these groups were lumped together for simplicity. DC density in breast cancers of different immunophenotype is presented in Table 4.

We investigated the correlations between populations of dendritic cells and Ki67 expression, tumour size and mitotic activity. Intratumoural CD1a+ cells showed a subtle positive correlation with Ki67 expression (R = 0.27, *p* < 0.001) and moderate with the mitotic figure count (R = 0.41, *p* < 0.001). By contrast, invasive margin CD1a+ cell number correlated slightly with mitotic activity exclusively (R = 0.22, *p* < 0.01). The intratumoural CD123+ cells showed subtle positive correlation with tumour size (R = 0.28, *p* < 0.001) as well as moderate positive correlation with Ki67 expression (R = 0.39, *p* < 0.001) and the mitotic figure count (R = 0.35, *p* < 0.001). Ki67 expression and mitotic activity also correlated positively with invasive margin CD123+ cell infiltration—weakly (R = 0.29, *p* < 0.001) and moderately (R = 0.39, *p* < 0.001), respectively. On the contrary, both intratumoural and invasive margin CD83+ cells inversely and weakly correlated with mitotic activity (R = −0.20, *p* < 0.02 and R = −0.18, *p* = 0.03, respectively). Additionally, intratumoural CD83+ cell infiltration inversely correlated with Ki67 expression (R = 0.18, *p* < 0.035).

### 3.3. DC Subpopulations and Other Pathological Prognostic Factors

With reference to tumour size, only intratumoural CD123+ DC count differed significantly (*p* < 0.015) between groups of different pT. The difference was significant between pT1 and pT2 patients (*p* < 0.015). The highest cell number was observed in pT2 (mean 22.57, SD 22.68), followed by pT1 (mean 16.39, SD 27.61) and pT3 tumours (mean 12.40, SD 15.53). Similarly, stratification according to the size into tumours of diameter ≤2 cm (pT1) and >2 cm (pT > 1) revealed a significant difference for intratumoural CD123+ staining (*p* < 0.009) exclusively, with a higher average number of positive cells in pT > 1 lesions (mean 21.65, SD 22.16) in comparison with pT1 tumours (mean 16.39, SD 27.61).

There were no statistically significant differences in DC count between cases with and without nodal involvement; the most pronounced differences between the groups of different pN were observed for intratumoural CD123+ (*p* < 0.15) and intratumoural DC-SIGN (*p* < 0.15) cell infiltration. Again, upon stratification into tumours with nodal metastases (pN+) and without nodal involvement (pN−), a significant difference was noted for intratumoural CD123+ cell count (*p* < 0.025) exclusively; the infiltration was higher in tumours of positive (22.81 SD 28.31) in comparison to those of negative nodal status (15.37 SD 20.75).

The population of intratumoural CD123+ cells was also the only one significantly associated with patients’ stage of disease (*p* < 0.004). The highest cell number was observed in stage II (mean 23.47, SD 29.95), followed by stage III (mean 21.97, SD 22.60) and stage I (mean 12.59, SD 20.38).

**Figure 2:**
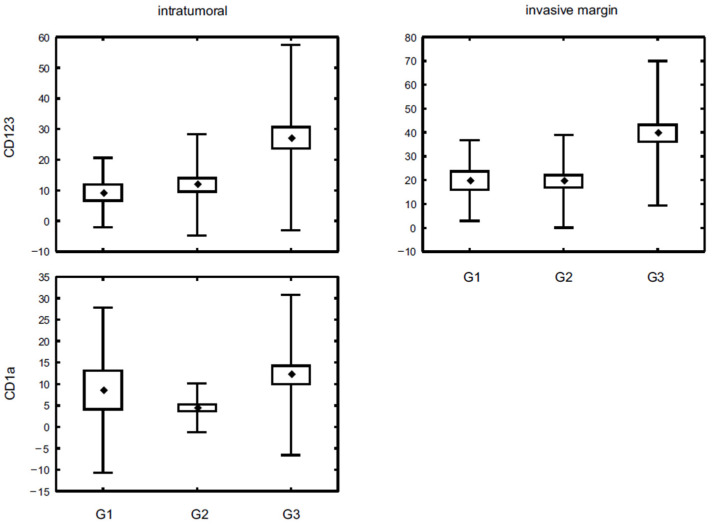
The density of CD123+ and CD1a+ DC populations in breast cancer specimens representing different nuclear grade. Central point is the arithmetic mean, box is the arithmetic mean ± standard error and whisker is the arithmetic mean ± standard deviation.

**Figure 3:**
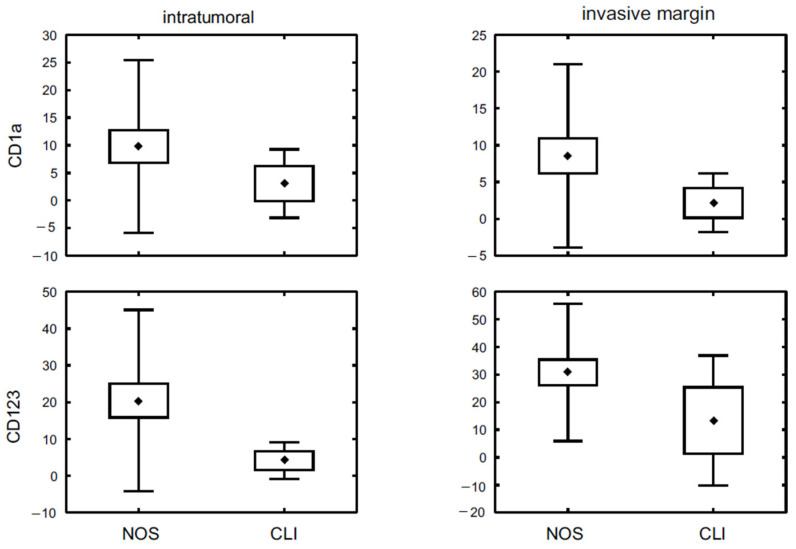
The density of CD1a+ and CD123+ DC populations in breast cancer specimens representing different histologic type: NOS—invasive carcinoma not otherwise specified, CLI—invasive lobular carcinoma. Central point is the arithmetic mean, box is the arithmetic mean ± 2 × standard error and whisker is the arithmetic mean ± 0.95 × standard deviation.

We have also investigated correlations with reference to tumour grade. A statistically significant difference was shown for intratumoural CD1a+ as well as intratumoural and invasive margin CD123+ cells. In both spatial locations of CD123+ cells, the highest cell density was observed in poorly differentiated tumours, which differed significantly in comparison with G2 (*p* < 0.001 for both intratumoural and invasive margin area) and G1 stage (*p* < 0.001 for intratumoural and *p* < 0.04 for invasive margin staining). Similarly, the most intense infiltration of intratumoural CD1a+ cells was associated with G3 tumours; however, it differed significantly only with G2 tumours (*p* < 0.015) (Figure 2, Table 5). In respect of tumour histological type, a higher count of CD1a+ and CD123+ DCs was observed in invasive carcinoma of no special type compared to invasive lobular carcinoma (Figure 3, Table 6).

### 3.4. Survival Analysis

In the investigated group, the data on clinical outcome were available for 100 patients. Out of them, distant metastases were observed in 18 cases (18%). The most frequent localizations were lymph nodes (7 cases, 39%), followed by bones and liver (6 cases, 33%), lungs (4 cases, 22%) and ovary (2 cases, 11%).

**Figure 4:**
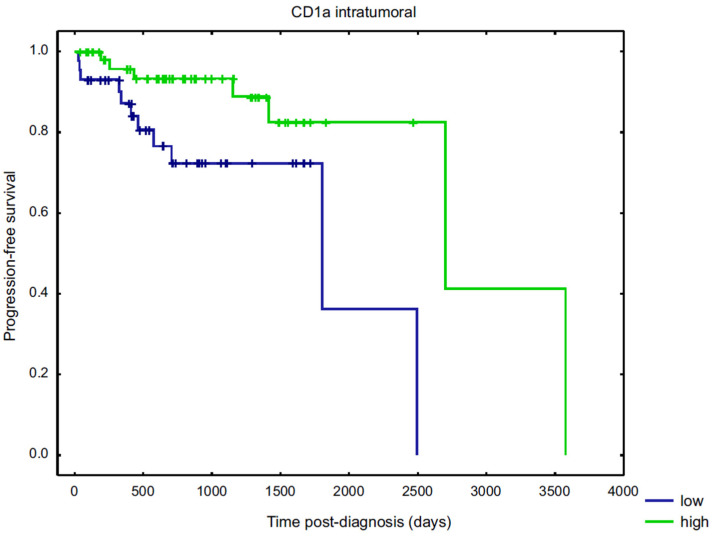
Kaplan–Meier curves for progression-free survival (PFS). Comparison of PFS according to intratumoural CD1a+ cell infiltration stratified by the median value of positively stained cells.

The number of individual subpopulations of dendritic cells was divided into “low” or “high” on the basis of median value (cell count ≤ median value was regarded as “low” and cell count > median value was regarded as “high”). For intratumoural and invasive margin CD83+ as well as intratumoural DC-SIGN+, “low” cell infiltration was determined if no cells were observed in five HPF. According to the Kaplan–Meier method, among investigated DC subpopulations, only intratumoural CD1a+ cells were significantly associated with PFS (*p* < 0.015), as the patients showing high DC count tended to have a longer PFS (in days) than patients with low infiltrated lesions (Figure 4).

Of the investigated variables, only pN significantly influenced the hazard ratio for breast cancer distant metastases’ incidence in the multivariate Cox regression model (*p* < 0.002). The patients with higher nodal involvement were at a higher risk of developing distant metastases (Hazard Ratio = 2.54, CI: 1.43–4.51). None of the analysed DC subpopulations had a significant impact on metastases development in this model.

### 3.5. DC Subpopulation Distribution

**Figure 5:**
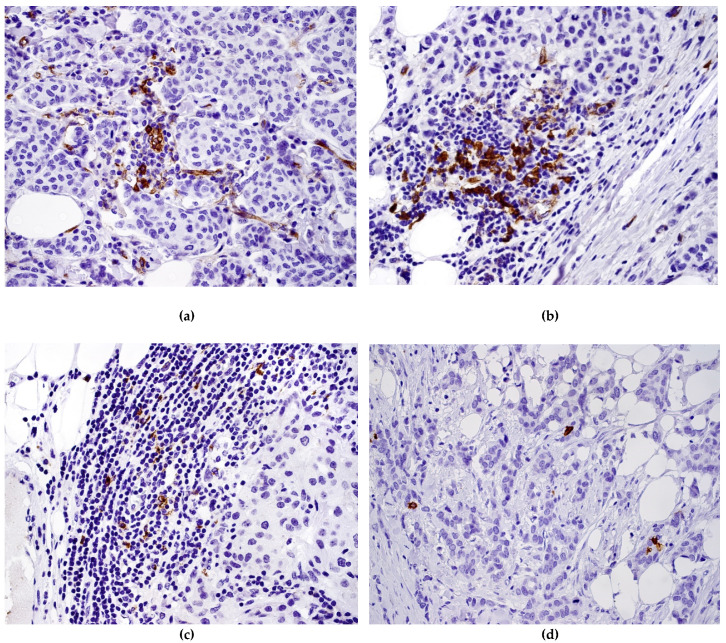
Dendritic cells in breast carcinoma: (**a**) intratumoural CD123+ cells, (**b**) invasive margin CD123+ cells, (**c**) LAMP3+ cells at invasive margin, (**d**) CD1a+.

The average count of positive DC count was 8.75 (SD 15.45) for intratumoural CD1a-positive (CD1a+), 7.77 (SD 12.54) for invasive margin CD1a+, 8.04 (SD 15.12) for intratumoural LAMP3-positive (LAMP3+), 11.25 (SD 12.65) for invasive margin LAMP3+, 19.12 (SD 25.20) for intratumoural CD123-positive (CD123+), 29.66 (SD 27.06) for invasive margin CD123+, 1.51 (SD 3.65) for intratumoural CD83-positive (CD83+), 3.10 (SD 6.15) for invasive margin CD83+, 5.55 (SD 11.20) for intratumoural DC-SIGN-positive (DC-SIGN+) and 13.0 (14.96) for invasive margin DC-SIGN+. The significantly higher DC densities were observed at the tumour’s invasive margin for LAMP3+, CD123+, CD83+ and DC-SIGN+ cells, in comparison with intratumoural area (*p* < 0.001 for each DC subpopulation). Examples of DC distribution are shown in Figure 5.

## 4. Discussion

The role of DCs in tumourigenesis had been studied in recent decades, which led researchers to the conclusion that the immune system plays a prominent role in tumour control. DCs, which appeared to respond to antigens as the first line of cells, play a central role in initiating antigen-specific immunity and tolerance. However, the predictive significance of tumour infiltrating DCs presenting a variety of different markers remains unresolved [15,23]. In our study, we made an attempt to establish the correlation between DC infiltrate density and histological and molecular characteristics of breast cancer.

Bell et al. failed to establish a prognostic significance of the infiltration of tumours with mature or immature DCs due to a limited number of samples (32 patients) [2], while we, based on 100 cases of breast cancer, have demonstrated that intratumoural CD1a+ cells were significantly associated with PFS. Patients with highly infiltrated tumours tended to have a longer PFS than patients with low infiltrated lesions. We think that an increase in CD1a+ cell count attracted to the tumour environment has a capability to initiate an immune response to the malignancy in the host organism, which results in a better prognosis. Coventry and Heinzel had proposed a possible explanation for this observation as they hypothesized that CD1a is an important molecule for the presentation of glycolipid tumour antigens to the immune system [15]. Coventry and Morton showed lower mortality rate of patients with breast cancer presenting with higher CD1a+ DC density within the tumour. Although they could not demonstrate a significant association with a 5-year survival due to sample size (51 patients), they postulated that an association between CD1a expression may reach statistical significance at the 10-year point from diagnosis [24]. La Rocca et al. have suggested a possible role of CD1a as a prognostic marker in breast cancer [8]. The density of tumour residing CD1a+ DCs has also been reported in a variety of human cancers, and their number in colon, gastric, lung and laryngeal carcinomas was positively associated with improved outcome [25,26,27,28].

CD1a+ DCs have been reported to be present within breast cancers from early, preinvasive ductal carcinoma in situ to invasive ductal carcinomas [9]. Bell et al. studied 32 cases of breast carcinoma and reported that immature DCs (CD1+, Langerin+) are found within the tumour bed, whereas mature DCs (CD83+, DC-LAMP+) reside in the peritumoural area [2]. Such a conclusion was reiterated by other researchers [9,10,24,29]. In our study, we have not observed statistically significant differences in the spatial distribution of CD1a+ DC subpopulation, but higher numbers of these cells were found mainly within the tumour. In contrast, mature DCs expressing LAMP3, CD123, CD83 and DC-SIGN were significantly more abundant at the invasive margin.

An association between the presence of CD123+ pDCs and shorter overall survival as well as relapse-free survival in patients with breast cancer was the main observation of the research by Treilleux et al. [10]. The results of a successive study by Sisirak et al. demonstrated a significantly higher density of tumour associated CD123+ pDCs in aggressive carcinomas such as triple negative breast tumour [11]. Our report seems to be consistent with such a conclusion, as we showed that pDCs preferentially and abundantly infiltrate these aggressive breast tumours. Although the triple negative molecular subtype is only found in about 15 percent of breast cancers, it is known for its aggressiveness, unresponsiveness to treatment and poor prognosis [30,31,32]. As the mechanism explaining the role of plasmacytoid DCs in tumour growth, Sisirak at al. proposed the interference in immune response toward immunotolerance caused by a defect in interferon alfa production by pDCs, which leads to Treg expansion in the tumour site and contributes to breast cancer progression [11].

Iwamoto et al. demonstrated that the infiltration of breast cancer by CD83+ DCs is an independent immunologic prognostic parameter, as the number of intratumoural CD83+ DCs was inversely correlated with lymph node metastasis and significantly associated with longer relapse-free and overall survival. Additionally, among patients with lymph node metastasis, the survival rate of those with larger numbers of CD83+ DCs intratumourally was significantly higher than that of patients with fewer CD83+ DCs. Such an observation for survival rates was not detected for the number of CD1a+ DCs [29]. We were not able to confirm these results, but we demonstrated denser CD83+ DC infiltrate in luminal tumours compared to non-luminal ones. These differences may result from the use of different methodology—in our study, immunohistochemistry was performed on formalin-fixed paraffin-embedded tissue, while in the study cited above, immunohistochemistry was based on frozen tissue.

A review of the literature revealed that little is still known about DCs in different molecular subtypes of breast cancer, thus their role should be clarified. Our study notes some significant differences in molecular subtypes of breast cancer and DC infiltration. To the aforesaid observations on triple negative breast tumours, we add that luminal/HER2+ tumours contained a greater number of CD1a positive DCs. To the best of our knowledge, this statement has never been mentioned before.

Several authors focused on investigating the presence of different subsets of DCs in breast cancer depending on tumour characteristics. Coventry and Morton and Treilleux et al. subsequently showed the lack of a significant correlation between CD1a+ cell density and classical histological or clinical prognostic variables [10,24] such as tumour size, grade, nodal status, presence or absence of metastases, recurrences or lymphovascular invasion. This seems to confirm a similar conclusion resulting from the preceding study by Iwamoto et al., except noticing an inverse correlation of the number of CD83+ DCs with lymph node metastases [29]. Researchers reported an association between DC-LAMP+ DC density and axillary lymph node involvement, high histologic grade, HER2 overexpression and lack of hormone receptor expression instead [10]. Gadalla et al. demonstrated increased infiltration of plasmacytoid DCs expressing CD303 in breast cancer tissue in patients with lymph node metastases compared to lymph node negative patients [33]. The results we obtained allow us to state that, considering classical histological variables, more advanced tumours (>pT1) as well as those with nodal involvement (pN+) were associated with higher plasmacytoid CD123+ DC count within the tumour mass. Moreover, in stage II and stage III tumours, as well as in poorly differentiated ones (G3), we have observed a more dense CD123+ DC infiltrate intratumourally. Additionally, in our investigation more abundant infiltrate composed of both intratumoural and peritumoural CD1a+ and CD123+ DCs was observed in NOS compared to CLI. Similarly to the results obtained by Iwamoto [29], we observed that the negative status of both ER and PR was related to LAMP3+ DC presence, which may contribute to tumour aggressiveness as well as resistance to tamoxifen therapy [34]. We also noticed that the analysed populations of DCs correlated positively with high Ki67 expression and HER2 overexpression/amplification. This supports the aforementioned hypothesis of intratumoural plasmacytoid CD123+ DC deleterious influence on tumour progression and explains their negative impact on patient survival [11,35].

The findings of this study have to be seen in the light of possible limitations. First, immunohistochemical staining allowed us to identify only one marker per slide; therefore, we could not observe the colocalization of investigated markers in tumour tissue nor in individual DCs. This hindered more precise identification of DC populations and their maturation state. The second limitation concerns the specificity of analysed DC-associated antigens, which can be found in other immune cells such as lymphocytes or macrophages. To overcome these difficulties, we took the cell appearance (e.g., nucleus size, the amount of cytoplasm and the presence of cytoplasmatic protrusions) into consideration in identifying positively stained cells as DCs. We also interpreted the results with caution and pointed to positivity for particular markers in DC populations rather than using the term “mature” or “immature” DCs. Another limitation concerns the type of material used in this study. Histological material presents a static image of the fixed tissue, while ex vivo models show dynamic processes and represent a valuable research resource which might improve understanding of the neoplastic process [36,37].

Besides their possible prognostic significance, DCs are also an attractive target to be exploited in cancer immunotherapy as they were shown to be dysfunctional in patients with breast cancer [38]. In recent years, the use of ex vivo manipulated [16], in vivo targeting DCs [39] and DCs in combination with cytokine-induced killer cells [3] has been regarded as a potential tool in cancer treatment with an already established role in prostate cancer therapy, in which the Food and Drug Administration has approved the first cancer DC-vaccine [40]. An increasing number of both preclinical and clinical evidence show that dendritic cell-based vaccines are capable of inducing an antitumour-specific response, while being well tolerated and safe [1,41]. Still, much has to be done in order to reach a satisfactory clinical outcome, but it seems to be a very promising future approach either as applied alone or in combination with traditionally used chemo- or radiotherapy [23].

## 5. Conclusions

In conclusion, our study indicates associations between DCs with tumour histological and clinical characteristics in breast carcinoma. We also showed differences between breast cancer molecular subtypes and their different DC lineage contents. However, many questions remain to be answered to elucidate their impact on prognosis in breast cancer patients.

## Figures and Tables

**Table 1:** Antibodies used in the study.

Primary Antibody	Clone	Dilution	Antigen Retrieval	Incubation Time	Producer
CD1a	MTB1	1:10	Citrate	Overnight	Novocastra (Leica Biosystems, Nußloch, Germany)
CD123	BR4MS	1:100	Ethylenediaminetetraacetic Acid Disodium (EDTA)	30 min	Novocastra (Leica Biosystems, Nußloch, Germany)
CD83	1H4b	1:50	Citrate	30 min	Abcam, Cambridge, UK
DC-LAMP3	Rabbit polyclonal	1:50	EDTA	60 min	Abcam, Cambridge, UK
DC-SIGN	5D7	1:50	EDTA	30 min	Abcam, Cambridge, UK
Estrogen receptor	6F11	1:25	Citrate	60 min	Novocastra (Leica Biosystems, Nußloch, Germany)
Progesterone receptor	PgR636	1:50	Citrate	60 min	Dako, Santa Clara, CA, USA
Ki67	MIB-1	1:100	EDTA	60 min	Dako, Santa Clara, CA, USA

**Table 2:** Clinicopathologic features of the study group.

Characteristic	Number of Cases	%
Age Mean: 54.9 Range: 29–87		
Stage		
I	51	33.5
II	58	38.2
III	40	26.3
IV	1	0.7
Unknown	2	1.3
Tumour size		
pT1	77	50.7
pT2	70	46.0
pT3	5	3.3
Lymph node status		
pN0	69	45.4
pN1	41	27.0
pN2	21	13.8
pN3	18	11.8
Unknown	3	2.0
Histological type		
Invasive carcinoma of no special type	133	87.5
Invasive lobular carcinoma	17	11.2
Other	2	1.3
Nottingham Histologic Grade		
G1	19	12.5
G2	57	37.5
G3	76	50.0
Molecular subtypes		
Luminal/HER2−	72	47.4
Luminal B/HER2+	27	17.8
Non-luminal HER2+	21	13.8
Triple negative breast cancer	30	19.7
Unclassified	2	1.3

**Table 3:** DC density in breast cancers of different molecular subtype.

	CD1a				LAMP3				CD123				CD83				DC-SIGN			
	intratumoural		invasive margin		intratumoural		invasive margin		intratumoural		invasive margin		intratumoural		invasive margin		intratumoural		invasive margin	
	mean (SD)	*p*	mean (SD)	*p*	mean (SD)	*p*	mean (SD)	*p*	mean (SD)	*p*	mean (SD)	*p*	mean (SD)	*p*	mean (SD)	*p*	mean (SD)	*p*	mean (SD)	*p*
Molecular subtype																				
Luminal/HER2−	7.55 (15.78)	<0.001	6.52 (12.07)	<0.001	3.21 (5.65)	<0.001	10.08 (10.54)	<0.001	10.35 (13.82)	<0.001	21.44 (20.83)	<0.009	2.69 (4.79)	<0.004	3.99 (5.59)	<0.05	4.02 (6.23)	NS	11.76 (11.28)	NS
Luminal B/HER2+	14.84 (17.84)		14.92 (18.88)		2.62 (6.81)		6.04 (13.67)		24.92 (22.85)		37.77 (33.03)		0.19 (0.57)		2.85 (10.52)		8.24 (15.21)		11.96 (14.69)	
HER2+ non-luminal	4.43 (4.80)		4.67 (5.89)		13.62 (14.00)		9.52 (9.54)		37.48 (44.16)		37.33 (32.41)		1.00 (2.36)		2.65 (3.77)		9.15 (19.43)		14.45 (12.66)	
Triple negative	10.0 (16.84)		7.33 (8.51)		20.10 (25.52)		19.30 (15.05)		22.30 (23.09)		37.10 (26.64)		0.33 (1.32)		1.77 (2.90)		3.63 (6.51)		14.06 (19.70)	

NS—not statistically significant, SD—standard deviation.

**Table 4:** DC density in breast cancers of different immunophenotype.

	CD1a				LAMP3				CD123				CD83				DC-SIGN			
	intratumoural		invasive margin		intratumoural		invasive margin		intratumoural		invasive margin		intratumoural		invasive margin		intratumoural		invasive margin	
	mean (SD)	*p*	mean (SD)	*p*	mean (SD)	*p*	mean (SD)	*p*	mean (SD)	*p*	mean (SD)	*p*	mean (SD)	*p*	mean (SD)	*p*	mean (SD)	*p*	mean (SD)	*p*
ER expression																				
positive	10.04 (17.75)	NS	8.95 (14.02)	NS	4.98 (14.19)	<0.001	9.37 (12.07)	0.001	15.19 (19.89)	<0.001	27.84 (26.91)	NS	1.98 (4.17)	NS	3.62 (6.92)	NS	5.33 (9.47)	NS	12.39 (12.41)	NS
negative	5.83 (6.50)		5.32 (7.46)		16.00 (15.01)		15.80 (13.32)		29.46 (34.09)		34.68 (27.62)		0.35 (1.07)		1.87 (3.30)		5.55 (14.38)		13.32 (17.87)	
PR expression																				
positive	10.30 (18.05)	NS	8.83 (14.24)	NS	5.42 (14.63)	<0.001	9.91 (12.27)	<0.03	15.0 (19.16)	<0.002	27.03 (25.97)	NS	1.97 (4.20)	NS	3.57 (7.01)	NS	4.87 (8.02)	NS	12.34 (11.88)	NS
negative	5.60 (6.23)		5.73 (7.50)		14.04 (14.94)		14.11 (13.40)		28.35 (33.92)		35.91 (29.17)		0.56 (1.66)		2.16 (3.47)		6.57 (15.88)		13.36 (18.27)	
Ki67 expression																				
low	6.51 (14.75)	<0.002	6.50 (12.54)	<0.02	5.64 (9.96)	NS	9.75 (9.98)	NS	11.35 (14.54)	<0.001	19.98 (19.74)	<0.001	2.35(4.74)	<0.04	3.64 (5.71)	NS	5.76 (13.49)	NS	12.29 (12.83)	NS
high	10.74 (15.98)		8.95 (12.61)		9.97 (18.12)		12.29 (14.45)		25.30 (30.03)		37.37 (29.78)		0.89 (2.35)		2.75 (6.52)		5.11 (8.74)		12.92 (15.03)	
HER2 status																				
normal	8.29 (16.06)	<0.03	6.77 (11.07)	<0.04	8.33 (16.61)	NS	12.92 (12.75)	<0.006	13.87 (17.82)	<0.001	26.09 (23.69)	NS	1.99 (4.23)	NS	3.32 (5.02)	NS	3.91 (6.29)	NS	12.46 (14.32)	NS
overexpressed	10.09 (14.41)		10.24 (15.24)		7.53 (11.88)		7.60 (12.01)		30.53 (34.23)		37.57 (32.40)		0.54 (1.64)		2.76 (8.21)		8.64 (17.01)		13.07 (13.73)	

NS—not statistically significant, SD—standard deviation.

**Table 5:** DC count according to tumour grade.

	CD1a				LAMP3				CD123				CD83				DC-SIGN			
	intratumoural		invasive margin		intratumoural		invasive margin		intratumoural		invasive margin		intratumoural		invasive margin		intratumoural		invasive margin	
	mean (SD)	*p*	mean (SD)	*p*	mean (SD)	*p*	mean (SD)	*p*	mean (SD)	*p*	mean (SD)	*p*	mean (SD)	*p*	mean (SD)	*p*	mean (SD)	*p*	mean (SD)	*p*
Grade																				
1	8.61 (19.25)	<0.009	6.77 (9.14)	NS	6.15 (9.38)	NS	10.16 (7.17)	NS	9.26 (11.37)	<0.001	19.84 (16.93)	<0.001	2.26 (4.36)	NS	3.37 (5.05)	NS	1.87 (3.48)	NS	9.19 (6.52)	NS
2	4.49 (5.69)		6.30 (7.21)		4.18 (7.39)		10.74 (12.21)		11.79 (16.56)		19.53 (19.49)		1.91 (4.06)		3.64 (5.58)		3.82 (5.39)		11.92 (11.72)	
3	12.10 (18.69)		9.15 (15.98)		11.29 (19.27)		11.91 (14.06)		27.20 (30.27)		39.71 (30.29)		1.03 (3.06)		2.63 (6.82)		7.66 (14.67)		14.62 (18.01)	

**Table 6:** DC density in breast cancers of different histologic type.

	CD1a				LAMP3				CD123				CD83				DC-SIGN			
	intratumoural		invasive margin		intratumoural		invasive margin		intratumoural		invasive margin		intratumoural		invasive margin		intratumoural		invasive margin	
	mean (SD)	*p*	mean (SD)	*p*	mean (SD)	*p*	mean (SD)	*p*	mean (SD)	*p*	mean (SD)	*p*	mean (SD)	*p*	mean (SD)	*p*	mean (SD)	*p*	mean (SD)	*p*
Histologic type																				
NOS	9.53 (16.18)	<0.015	8.57 (13.11)	<0.002	8.83 (15.91)	NS	12.08 (13.14)	NS	20.87 (26.12)	<0.001	30.51 (26.27)	<0.001	1.24 (3.08)	NS	2.94 (6.12)	NS	5.77 (11.82)	NS	13.32 (15.58)	NS
Lobular	3.06 (6.53)		2.18 (4.20)		2.41 (4.09)		6.00 (6.94)		4.12 (5.25)		13.35 (24.74)		3.71 (6.33)		4.65 (6.63)		4.18 (4.93)		10.29 (9.56)	

## Data Availability

The data presented in this study are available on request from the corresponding author. The data are not publicly available due to personal data protection.
